# Factors Affecting Compliance of Infection Control Measures among Dental Radiographers

**DOI:** 10.1155/2020/8834854

**Published:** 2020-11-16

**Authors:** Maram Alakhras, Dana S. Al-Mousa, Arwa Mahasneh, Amani G. AlSa'di

**Affiliations:** ^1^Faculty of Applied Medical Sciences, Jordan University of Science and Technology, Ar Ramtha, Jordan; ^2^Jordan Food and Drug Administration, Irbid, Jordan

## Abstract

**Objectives:**

To assess the level of dental radiographers' compliance with infection control measures and to evaluate the factors affecting their compliance.

**Methods:**

The study included 175 dental radiographers. Compliance with infection control was evaluated with a self-administered questionnaire consisting of 33 questions related to vaccination, hand hygiene (HH), personal protective equipment (PPE), disinfection and sterilization, and use of surface barriers. Pearson's chi-square test was used to compare compliance between subgroups of radiographers.

**Results:**

64.6% of participants were females, and 62.9% was younger than 30 years. 13.0% of the sample population had >10 years of experience and 28.0% take radiographs for >20 patients/day. 66.9% of participants wash their hands before/after taking radiographs. 26.3% of participants had vaccination against hepatitis B, tetanus, and tuberculosis. 12.6% fully use PPE, 10.9% perform complete disinfection and sterilization, and 16.0% apply all kinds of surfaces barriers. Vaccination was significantly affected by age, gender, and practice type. HH was affected by years of experience and number of patients radiographed per day. PPE was influenced by number of hours worked per week and patients radiographed daily. Disinfection and sterilization was affected by practice type and years of experience. The use of surface barriers was affected by age, practice type, and number of patients radiographed/day.

**Conclusions:**

The current study indicated poor compliance with infection control practices among dental radiographers. We recommend continuing educational programs and training courses to increase dental radiographers' awareness of local and international infection control guidelines and to enhance their implementation of these guidelines.

## 1. Introduction

Radiographers working in dental clinics are at a high risk for acquiring a wide range of organisms through contact with potentially infectious blood or saliva. These include hepatitis B virus (HBV), hepatitis C virus (HCV), human immunodeficiency virus (HIV), tuberculosis, and upper respiratory infections [1]. Malpractice in dental radiography may cause disease transmission between patients and dental care workers. International associations of infection control, such as the American Dental Association (ADA), recommend some guidelines that should be implemented in dental clinics and applied by clinical staff.

Cross-contamination in dental radiology is of a great concern, particularly when intraoral radiographs are used because intraoral films are a source of contamination for the radiographic processor resulting in cross-contamination of subsequent films which pose a threat to subsequent patients [2, 3]. Also other areas in dental radiology operatory, such as X-ray cone and control panel, may become contaminated during X-ray imaging procedure [2, 4, 5].

General infection control procedures used in healthcare systems including vaccination, hand hygiene (HH), the use of personal protective equipment (PPE), disinfection and sterilization, and the use of surface barriers are mostly applicable to dental radiology. The Center for Disease Control and Prevention listed and highlighted the importance of applying these procedures in dentistry [6].

Vaccination is considered as the most effective method against infectious diseases of healthcare workers. However, most patients are not similarly protected which may increase the risk of transmission of infectious diseases and necessitate the use of other measures such as PPE [7]. PPE such as gown, gloves, face mask, and protective eyewear are considered effective means of preventing the transmission of blood borne viruses [8, 9]. Although sterile gloves may protect the technician, small defects in gloves can harm the patient if proper HH was not performed. Handwashing was reported to be effective in elimination of more than 99% of bacteria [10]. Disinfection and sterilization of items used in dental radiography including the X-ray tube head and arm rest, control panel and exposure control knob, aprons, thyroid collars, film holders, and positioning devices are also vital in preventing disease transmission [11]. While radiographers tend to use disinfection more often, it must be noted that even after disinfection, live bacterial and viral particles can still survive which emphasizes the need of sterilization whenever its possible [12].

In intraoral digital radiography, unlike X-ray films, the same digital image detector is used for all patients but cannot be heat-sterilized [13]. This necessitates the use of protective barriers to cover the digital detectors in order to prevent contamination.

Guidelines of infection control are well established in many parts of the world; however, compliance with these guidelines is still of a concern. Many previous studies focused on the importance of infection control in dental radiography worldwide [2–4, 11–15]. However, few studies evaluated the compliance of dental radiographers of infection control measures [11, 13, 16], and these studies were limited to the evaluation of the use of protective barriers. To our knowledge, none of these studies has examined the compliance of dental radiographers with universal and local infection control measures in detail. The aim of the current study is to assess the level of dental radiographers' compliance with vaccination, HH, PPE, disinfection and sterilization, and use of surface barriers and its association with sociodemographic characteristics.

## 2. Materials and Methods

This study was reviewed and approved by the human research ethics committee at Jordan University of Science and Technology (grant number 305-2018). Written informed consent was obtained from all participants.

### 2.1. Instrument

Self-administered anonymous questionnaire was used in the current study. The questionnaire consisted of two main parts. The first part covered sociodemographic and professional characteristics including, age, gender, level of education, practice type, years working as a dental radiographer, and the radiographers' workload in terms of hours worked per week and number of patients seen per day.

The second part consisted of 33 questions distributed into 5 parts related to the radiographers' practice and compliance with vaccination (4 questions), HH (4 questions), PPE (7 questions), disinfection and sterilization (14 questions), and the use of surface barriers (4 questions). Questions related to vaccination were answered by yes, no, or not sure responses. The response to the other parts used a 4-point Likert scale (never to always). The survey questions were adapted from previous research [17, 18]. Data entry was reviewed by random checking of 10% of entered information.

### 2.2. Target Facilities and Study Design

The study was conducted in north and center regions of Jordan between January and April 2019. A convenient sample from clinics which have dental radiography facility in these regions was involved including nine major public hospitals and centers, two university affiliated hospitals, and one hundred private dental clinics/centers. All dental radiographers in targeted facilities were invited to participate in the study. Two research assistants gathered questionnaire data by face-to-face interviews without tracking of who responded and who did not to ensure anonymity. A total of 300 questionnaires were distributed, of which 175 radiographers successfully returned complete questionnaires resulting in a response rate of 58.3%.

### 2.3. Data Analysis

Data were entered and analyzed by the Statistical Package for Social Sciences (SPSS) software version 11.0 (SPSS®: Inc., Chicago, IL, USA). Means, standard deviations, frequencies, and percentages were produced. The level of compliance of infection control practice was categorized into three subgroups and expressed as excellent, intermediate, and poor compliance according to the percentage of correct answers for all questions together and for each infection control measure. A score of 40% or less was classified as a poor compliance, 40%–70% was classified as an intermediate compliance, and a score of 70% or more was classified as an excellent compliance. The responses “always” and “yes” were considered as correct answers. Pearson's chi-square test was used to compare between subgroups. The level of significance was set at *P* ≤ 0.05.

## 3. Results

Table 1 shows the demographic and professional characteristics of the study population.

The results about the behavior of participants toward vaccination, HH, PPE, disinfection and sterilization, and surface barriers are shown in Table 2. About one quarter of participants reported that they were vaccinated against all previously mentioned diseases. Table 2 also shows that the majority (66.9%) of respondents wash their hands before and after taking radiographs. Regarding PPE, the results found that 12.6% of respondents were with full compliance in using all PPE. Wearing masks, head cover, and protective eyewear were applied less frequently.

Only 10.9% of respondents perform frequent and complete disinfection and sterilization for all surfaces, equipment, and devices. Noting, the routine wiping by using surface disinfection was the most frequent reported behavior, while the disinfection of thyroid collar was the lowest (58.9% and 38.3%, respectively).

In relation to the use of surface barriers, Table 2 shows that only 16.0% of respondents apply all types of barriers while performing a dental radiograph.

**Figure 1:**
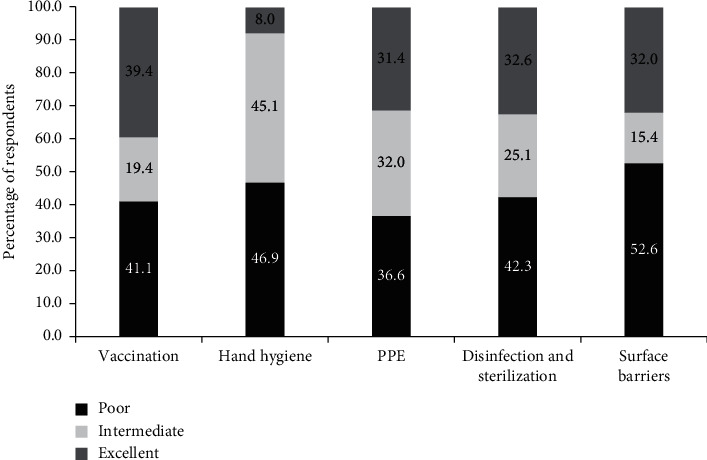
The level of compliance with infection control measures for all participants (*N* = 175).

Figure 1 shows the level of compliance of infection control practice among study participants. 39.4% of respondents had an excellent level of compliance with vaccination and 8.0% with HH. Approximately the same percentage of respondents (around 30%) showed excellent compliance with PPE, disinfection and sterilization, and the use of surface barriers. More than 50% of participants had a poor compliance in using surface barriers.

**Figure 2:**
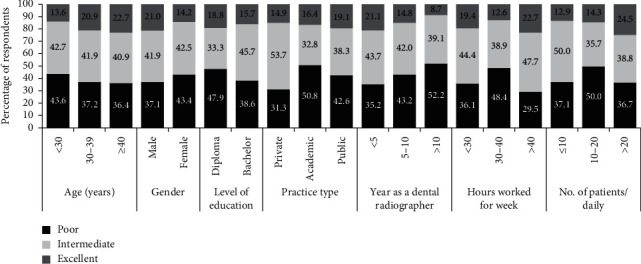
The overall level of compliance with infection control measures for subgroups of participants according to sociodemographic and professional characteristics (*N* = 175).

Figure 2 indicates the overall level of compliance of each subgroup of participants. Excellent level of compliance ranged from 8.7% for radiographers working for more than 10 years as a dental radiographer and 24.5% for radiographers who radiograph more than 20 patients per day. While the poor level of compliance ranged from 29.5% for radiographers working for more than 40 hours per week to 52.2% for radiographers who perform more than 10 images per day. The chi-square test showed no significant difference in the overall level of compliance between subgroups of respondents.

Comparison of the level of compliance with vaccination practices between groups of radiographers according to sociodemographic and background characteristics are demonstrated in Table 3. About 73.0% of respondents who are ≥40 years of age had a significantly higher excellent level of compliance toward vaccination compared with other age groups. Also, 53.2% of male respondents were significantly more compliant with vaccination practice in comparison with 31.9% of females. The percentage of compliant dental radiographers working in academic institutions to vaccination (31.1%) was significantly lower than those who work in private and public sectors (41.8% and 46.8%, respectively).

Table 4 reports the factors that may affect dental radiographers' level of compliance with HH. It shows that radiographers who have more than 10 years of experience as a dental radiographer had a significantly less compliance to HH compared with less experienced radiographers. Radiographers who radiograph more than 20 patients daily had a higher percentage of excellent compliance to HH compared with that of radiographers who radiograph a smaller number of patients daily.

About 53% of respondents who work for less than 30 hours weekly were more committed to using PPE compared with those who work for more than 30 hours weekly (Table 5). Also, radiographers who perform dental radiographs for less than 10 patients in a day had a higher compliance to using PPE in comparison with those who radiograph more than 10 patients daily.

The results showed that the level of compliance of practicing sterilization and disinfection by radiographers in private clinics was significantly better than the level of compliance of those who work at public and academic institutions. Also, radiographers who have less than 5 years of experience showed significantly greater compliance in disinfection and sterilization compared with their former counterparts (Table 6).

The factors affecting the radiographers' level of compliance with using surface barriers are shown in Table 7. Radiographers who are 30–40 years of age were significantly better in using surface barriers in comparison with those younger and older ones. As well as, workers at public hospitals showed higher compliance in using surface barrier compared with private and academic institutions. Moreover, radiographers who image more than 20 patients per day reported that they use surface barriers more than those who radiograph less numbers daily.

## 4. Discussion

To the best of our knowledge, this is the first study to assess the level of dental radiographers' compliance with all infection control measures. The results of the current study indicated that the general practice of infection control of respondents can be described as poor, based on percentages of 10.9%–26.3% of radiographers who completely apply all infection control measures in each domain with the worst scoring domain being the full application of disinfection and sterilization (10.9%). This result is consistent with the findings of previous studies performed in Australia [19] and Japan [20], which reported poor compliance of radiographers with infection control practices.

Regarding the level of compliance for all participants, the present research indicated that the percentage of participants who had poor compliance with each infection control measure was higher than those with intermediate and excellent compliance. Vaccination had the highest percentage of excellent level of compliance, while HH was the lowest among the other infection control practices.

The results also showed that the overall level of compliance of infection control practices was not significantly influenced by any sociodemographic or professional characteristic of respondents which corresponds to the results from a previous study [17]. However, specific infection control domains were significantly affected by different sociodemographic and professional characteristics. The factors affecting each domain will be discussed separately in the following sections.

### 4.1. Vaccination

All dental care practitioners are susceptible to transmission of infections and should be immunized against common diseases as recommended by many organizations including the American Dental Association 1996 [21] and the British Dental Association 2003 [22].

This study reported low to moderate level of compliance to immunization, with 69.1% of participants who had vaccination against hepatitis B, whilst lower proportions of participants reported that they are vaccinated against tetanus (44.0%) and tuberculosis (47.4%). Only 26.3% of study participants were vaccinated against all mentioned diseases. There is a variation in the level of compliance to vaccination among healthcare workers in the literature with some studies that reported higher rates of compliance to vaccination (89.9% [23] and 86% [24]), while others reported lower rates of 48.9% [25] and 57.7% [26] than the present research. This might be due to the availability and ease of access to information from guidelines and courses which are the main predictors of compliance to vaccination as reported previously [7]. In addition, it must be acknowledged that in Jordan, while it is highly recommended to acquire hepatitis B vaccination, there is no legislation mandating it.

The level of compliance of vaccination was significantly associated with gender, with male participants showing better compliance than female counterparts. Also, older respondents were more likely to be vaccinated against infectious diseases than younger respondents. These results contradict the findings of a study on dentists by McCarthy and MacDonald [16] which found that women and younger participants were more likely to report HBV vaccination than men and older participants. The effect of gender might be attributable to uneven distribution of participants as 64.6% were females. Higher compliance of older participants can be due to the level of their knowledge gained with age, which is an important reason for vaccine compliance as reported previously [26]. Further work should be carried out to assess the correlation between knowledge and practice as well as reasons for vaccine noncompliance.

In addition to age and gender, radiographers working at private and public sectors had a higher level of compliance than radiographers working at academic institutions. There is no clear reason for this result, and further analysis is needed to explore other characteristics of radiographers working at academic institutions contributing to this difference.

### 4.2. Hand Hygiene

Hand contact with contaminated surfaces may transfer pathogens to patients or dental technicians. Hand contamination can cause microorganisms to be transmitted to equipment, other environmental surfaces, and patient's or technician's eyes, nose, or mouth. This highlights the importance of HH in preventing disease transmission.

The primary method usually used for hand cleansing is washing with soap and water. However, the HH guidelines published by “Centers for Disease Control and Prevention” (CDC) included the use of an alcohol-based hand sanitizer as a replacement to traditional hand washing for all patient contacts except if hands are clearly soiled [6]. It has been reported by CDC that the use of gloves helps protect patients and health practitioners and reduces the risk of contamination by 70%–80% [6]. However, wearing gloves may provide a suitable warm and moist environment for organisms; so, HH is essential to decrease cross-contamination even when gloves are worn. Our data showed that 66.9% of radiographers use washing, 46.9% use alcohol-based hand sanitizers for HH, and 50.9% of dental radiographers wash hands before wearing gloves. Educational programs on HH should be implemented in order to increase the level of compliance. A study by O'Donoghue et al. showed that the use of educational intervention increased the level of compliance from 28.9% preintervention to 51.4% postintervention [27].

The current study found that radiographers who have less experience as a dental radiographer tend to significantly have more adherences to HH practice than more experienced radiographers. This aligns with the results of a previous study which reported that recently graduated practitioners are more likely to adhere to infection control measures [28]. In contrary, other studies found that the higher experience level is associated with higher adherence to infection control [29], while others reported that the clinical experience does not affect the level of adherence to infection control [30].

Radiographers with higher workload in terms of number of patients radiographed daily had significantly higher compliance to HH than radiographers with less workload. This may be due to seeing the higher volume of patients which requires frequent HH.

While proper HH should be performed even with wearing gloves, it must be noted here that radiographer's compliance with HH was expected to be affected by their adherence to wear gloves. Therefore, additional analysis was performed to test the association between HH and PPE statements related to wearing gloves (wearing gloves while taking radiographs, wearing gloves while handling film packets, and changing gloves between patients). The analysis showed that higher compliance with HH items was associated with higher compliance with wearing/changing gloves. This means that even if the radiographers performed HH more frequently, they also complied to wear gloves.

### 4.3. Personal Protective Equipment

Wearing gloves, eye wear, face masks, and head covering are all means of reducing the radiographers' exposure to pathogens. Previous work has reported that 56%–100% of dental practitioners wore gloves, 32%–90.1% wore face masks, and 14.7%–91.2% wore eye protective wear [29]. The percentages found in the present research fall within these ranges with 61.1%, 45.7%, and 33.1% wore gloves, face masks, and eyewear, respectively. The results also found that only 12.6% of respondents were with full compliance in using all PPE where wearing gloves either while handling film packets or taking radiographs and changing gloves between patients were reported to be the most frequently committed behaviors by respondents (68.0%, 61.1%, and 53.1%, respectively). Wearing masks (45.7%), head cover (34.9%), and protective eyewear (33.1%) were applied less frequently. This is consistent with the results of a previous study which reported that gloves are the most used item while eye protection is the least used [8].

As indicated by our results, PPE practice was affected by the radiographers' workload in terms of the number of working hours per week and the number of imaged patients per day. Radiographers who work for less than 30 hours weekly and who radiograph ten or less patients daily were more committed to using PPE compared with those who work for longer than 30 hours per week and who image more than 10 patients each day. While examining higher number of patients was expected to be accompanied with more frequent use of PPE, the increase in workload [31, 32] and fatigue [33] associated with application of PPE might be reasons for noncompliance with PPE as indicated by other studies. Other contributing reasons might be the lack of discomfort, poor-quality of PPE, and lack of training about how to use PPE [33].

### 4.4. Disinfection and Sterilization

Equipment used in radiography can come in contact with patients' saliva or blood and act as reservoirs for cross infection. This equipment includes tube head, image receptors [34], thyroid collars [18], and lead aprons [35]. The Department of Health recommended that all hospital equipment used for more than one patient should be cleaned after each use [36]. It must be acknowledged that even after disinfection, live bacterial and viral particles can still survive [12] which emphasizes the need of sterilization whenever possible. The results of the current study indicated that the practice of disinfection and sterilization of devices used in imaging was generally “poor” with only 10.9% of respondents who fully disinfect or sterilize surfaces, equipment, and devices. Thyroid collars and lead aprons were among the lowest percentages which align with the results of other studies [18].

Radiographers working in private clinics reported a higher level of compliance to disinfection and sterilization practice than the ones who work at public and academic centers/clinics.

Also, radiographers with less experience showed better compliance than radiographers with more experience. Several systematic reviews reported that healthcare practitioners with less experience tend to apply clinical guidelines more than practitioners with more experience [37].

Similar to HH and use of surface barriers, radiographers who examined more than 20 patients per day had a higher compliance with disinfection and sterilization procedures compared with radiographers seeing ≤10 or 11–20 patients/day. However, this difference was not statistically significant (*P* value = 0.068).

### 4.5. Use of Surface Barriers

The ideal way to protect the digital image receptors is by using surface barriers because they are sensitive to disinfectants and cannot be sterilized by autoclaves [38]. In the present research, the percentage of radiographers covering digital receptors with protective barriers was 51.4%, which is lower than the percentage reported by other studies (72.0% for dentists and 90.2% for students) [18]. The percentage of radiographers who reported that they always use barrier protection for bite guide was only 44.0%, which can be partially explained by the difficulty of positioning or patient discomfort when they are used as suggested by other studies [20].

Our data indicated that middle age participants (30–40 years) had higher use of surface barriers than younger or older participants. Radiographers who work at public hospitals use surface barriers more than radiographers who work at private and academic centers/clinics. This can be explained by the increase in cumulative instruction to follow infection control procedures in public setting compared to smaller practices due to the presence of higher number of practitioners [30].

While using surface barriers for bite guards may cause patient discomfort, the overall use of surface barriers including bite guide, dental unit surfaces, digital sensors, and monitors was higher when imaging higher number of patients as shown by the current study. Radiographers who radiograph more than 20 patients indicated higher use of surface barriers (49.0%) than radiographers who image a smaller number of patients (22.9% and 28.6% for the groups who image ≤10 and 11–20 patients, respectively).

There are some limitations of the current study. Approximately 40% of the radiographers invited to participate failed to return a completed questionnaire, which may have led to nonresponse bias. The survey did not include questions related to the reasons of noncompliance with individual infection control measures which may help in clarification of some of the inconsistent findings.

## 5. Conclusion

To our knowledge, this is the first study that provides a comprehensive assessment of infection control practices including vaccination, HH, PPE, disinfection and sterilization, and use of surface barriers among dental radiographers. The findings of the present research demonstrate poor compliance with infection control practices among respondent radiographers. The results found that the sociodemographic and professional factors affecting compliance with infection control are not consistent among all five measures, and sometimes, it is not completely clear why specific groups of radiographers were less compliant than other subgroups. It is necessary to improve the radiographers' implementation of recommended infection control guidelines in dental radiography. This requires better understanding of the reasons behind noncompliance necessary to provide informed recommendations to academic and dental institutions to develop educational courses and continuing education programs about infection control.

## Figures and Tables

**Table 1:** Demographic and professional characteristics of study participants (*N* = 175).

Characteristic	*N* (%)
Age (years)	
<30	110 (62.9)
30–39	43 (24.6)
≥40	22 (12.6)
Gender	
Male	62 (35.4)
Female	113 (64.6)
Educational level	
Diploma or less	48 (27.4)
Bachelor or higher	127 (72.6)
Practice type	
Private clinics	67 (38.3)
Academic institutions	61 (34.9)
Public hospital/centers	47 (26.9)
Years as a dental radiographer	
<5	71 (40.6)
5–10	81 (46.3)
>10	23 (13.1)
Hours worked per week	
<30	36 (20.6)
30–40	95 (54.3)
>40	44 (25.1)
Number of patients per day	
≤10	70 (40.0)
11–20	56 (32.0)
>20	49 (28.0)

**Table 2:** Radiographers' practice regarding infection control measures (*N* = 175).

	Yes *N* (%)	No *N* (%)	Not sure *N* (%)
Vaccination			
Vaccination against hepatitis B	121 (69.1)	42 (24.0)	12 (6.9)
Booster shot	82 (46.9)	74 (42.3)	19 (10.9)
Vaccination against tetanus	77 (44.0)	65 (37.1)	33 (18.9)
Vaccination against TB	83 (47.4)	63 (36.0)	29 (16.6)
Full vaccination	**46 (26.3)**		
	Always *N* (%)	Occasionally *N* (%)	Rare/Never *N* (%)
Hand hygiene			
Washing hands B/A taking radiographs	117 (66.9)	41 (23.4)	17 (9.7)
Washing hands before wearing gloves	89 (50.9)	49 (28.0)	37 (21.1)
Using alcohol-based hand rubs instead of washing	82 (46.9)	49 (28.0)	44 (25.1)
Washing hands with antiseptic solution*∗*	30 (17.1)		
Personal protective equipment			
Wearing gloves while taking radiographs	107 (61.1)	42 (24.0)	26 (14.9)
Wearing gloves while handling film packets	119 (68.0)	31 (17.7)	25 (14.3)
Changing gloves between patients	93 (53.1)	54 (30.9)	28 (16.0)
Wearing protective eyewear	58 (33.1)	41 (23.4)	76 (43.4)
Wearing masks	80 (45.7)	38 (21.7)	57 (32.6)
Changing mask between patients	68 (38.9)	50 (28.6)	57 (32.6)
Using head covering	61 (34.9)	29 (16.6)	85 (48.6)
Fully apply PPE	**22 (12.6)**		
Disinfection and sterilization			
Using surface disinfection for routine wiping	103 (58.9)	35 (20.0)	37 (21.1)
Disinfection of tube head	85 (48.6)	51 (29.1)	39 (22.3)
Disinfection of tube arm rest	91 (52.0)	48 (27.4)	36 (20.6)
Disinfection of chair control	92 (52.6)	47 (26.9)	36 (20.6)
Disinfection of control panel	93 (53.1)	51 (29.1)	31 (17.7)
Disinfection of lead apron	78 (44.6)	46 (26.3)	51 (29.1)
Disinfection of thyroid collar	67 (38.3)	49 (28.0)	59 (33.7)
Disinfection of working area/counter tops	89 (50.9)	44 (25.1)	42 (24.0)
Sterilization of film holder	86 (49.1)	52 (29.7)	37 (21.1)
Sterilization of positioning device	86 (49.1)	52 (29.7)	37 (21.1)
Sterilization of bite guide	77 (44.0)	71 (40.6)	27 (15.4)
Cleaning the chin rest	76 (43.4)	69 (39.4)	30 (17.1)
Cleaning the head positioning guide	76 (43.4)	70 (40.0)	29 (16.6)
Cleaning the handgrips	78 (44.6)	67 (38.3)	30 (17.1)
Full application of disinfection and sterilization	**19 (10.9)**		
Surface barriers			
Using barrier protection for bite guide	77 (44.0)	62 (35.4)	36 (20.6)
Using surface barriers for dental unit surfaces	89 (50.9)	37 (21.1)	49 (28.0)
Using plastic barriers for digital sensors	90 (51.4)	54 (30.9)	31 (17.7)
Using plastic barriers for monitors	85 (48.6)	57 (32.6)	33 (18.9)
Full use surface barriers	**28 (16.0)**		

^*∗*^The responses to this question were yes or no. Bold values represent the full application of infection control measures.

**Table 3:** The level of compliance with vaccination practices (*N* = 175).

Characteristics	Vaccination level of compliance			Value of the *χ*^2^ test *P* value
	Poor *N* (%)	Intermediate *N* (%)	Excellent *N* (%)	
Age (years)				
<30	50 (45.5)	26 (23.6)	34 (30.9)	14.66
30–39	18 (41.9)	6 (14.0)	19 (44.2)	**0.005**
≥40	4 (18.2)	2 (9.1)	16 (72.7)	
Gender				
Male	20 (32.3)	9 (14.5)	33 (53.2)	7.67
Female	52 (46.0)	25 (22.1)	36 (31.9)	**0.022**
Educational level				
Diploma or less	20 (41.7)	9 (18.8)	19 (39.6)	0.020
Bachelor or higher	52 (40.9)	25 (19.7)	50 (39.4)	0.990
Practice type				
Private clinics	33 (49.3)	6 (9.0)	28 (41.8)	17.46
Academic institutions	20 (32.8)	22 (36.1)	19 (31.1)	**0.002**
Public hospital	19 (40.4)	6 (12.8)	22 (46.8)	
Years as a dental radiographer				
<5	32 (45.1)	14 (19.7)	25 (35.2)	1.88
5–10	32 (39.5)	14 (17.3)	35 (43.2)	0.758
>10	8 (34.8)	6 (26.1)	9 (39.1)	
Hours worked for week				
<30	14 (38.9)	10 (27.8)	12 (33.3)	6.16
30–40	35 (36.8)	20 (21.1)	40 (42.1)	0.188
>40	23 (52.3)	4 (9.1)	17 (38.6)	
Number of patients per day				
≤10	26 (37.1)	21 (30.0)	23 (32.9)	8.49
11–20	24 (42.9)	7 (12.5)	25 (44.6)	0.075
>20	22 (44.9)	6 (12.2)	21 (42.9)	

Bold values are the statistically significant differences.

**Table 4:** The level of compliance with HH practices (*N* = 175).

Characteristics	Hand hygiene level of compliance			Value of the *χ*^2^ test *P* value
	Poor *N* (%)	Intermediate *N* (%)	Excellent *N* (%)	
Age (years)				
<30	53 (48.2)	51 (46.4)	6 (5.5)	6.01
30–39	16 (37.2)	22 (51.2)	5 (11.6)	0.199
≥40	13 (59.1)	6 (27.3)	3 (13.6)	
Gender				
Male	32 (51.6)	25 (40.3)	5 (8.1)	0.958
Female	50 (44.2)	54 (47.8)	9 (8.0)	0.619
Educational level				
Diploma or less	23 (47.9)	22 (45.8)	3 (6.3)	0.276
Bachelor or higher	59 (46.5)	57 (44.9)	11 (8.7)	0.871
Practice type				
Private clinics	32 (47.8)	32 (47.8)	3 (4.5)	5.43
Academic institutions	33 (54.1)	23 (37.7)	5 (8.2)	0.246
Public hospital	17 (36.2)	24 (51.1)	6 (12.8)	
Years as a dental radiographer				
<5	27 (38.0)	37 (52.1)	7 (9.9)	9.59
5–10	38 (46.9)	36 (44.4)	7 (8.6)	**0.048**
>10	17 (73.9)	6 (26.1)	0 (0.0)	
Hours worked for week				
<30	17 (47.2)	17 (47.2)	2 (5.6)	1.17
30–40	46 (48.4)	42 (44.2)	7 (7.4)	0.883
>40	19 (43.2)	20 (45.5)	5 (11.4)	
Number of patients per day				
≤10	36 (51.4)	33 (47.1)	1 (1.4)	16.96
11–20	30 (53.6)	23 (41.1)	3 (5.4)	**0.002**
>20	16 (32.7)	23 (46.9)	10 (20.4)	

Bold values are the statistically significant differences.

**Table 5:** The level of compliance with PPE practices (*N* = 175).

Characteristics	PPE level of compliance			Value of the *χ*^2^ test *P* value
	Poor *N* (%)	Intermediate *N* (%)	Excellent *N* (%)	
Age (years)				
<30	45 (40.9)	30 (27.3)	35 (31.8)	4.312
30–39	14 (32.6)	16 (37.2)	13 (30.2)	0.365
≥40	5 (22.7)	10 (45.5)	7 (31.8)	
Gender				
Male	25 (40.3)	18 (29.0)	19 (30.6)	0.652
Female	39 (34.5)	38 (33.6)	36 (31.9)	0.722
Educational level				
Diploma or less	19 (39.6)	16 (33.3)	13 (27.1)	0.598
Bachelor or higher	45 (35.4)	40 (31.5)	42 (33.1)	0.741
Practice type				
Private clinics	17 (25.4)	22 (32.8)	28 (41.8)	8.779
Academic institutions	22 (46.8)	18 (29.5)	18 (29.5)	0.067
Public hospital	25 (41.0)	16 (34.0)	9 (19.1)	
Years as a dental radiographer				
<5	23 (32.4)	23 (32.4)	25 (35.2)	2.58
5–10	33 (40.7)	27 (33.3)	21 (25.9)	0.630
>10	8 (34.8)	6 (26.1)	9 (39.1)	
Hours worked for week				
<30	9 (25.0)	8 (22.2)	19 (52.8)	13.12
30–40	40 (42.1)	28 (29.5)	27 (28.4)	**0.011**
>40	15 (34.1)	20 (45.5)	9 (20.5)	
Number of patients per day				
≤10	22 (31.4)	17 (24.3)	31 (44.3)	12.44
11–20	21 (37.5)	18 (32.1)	17 (30.4)	**0.014**
>20	21 (42.9)	21 (42.9)	7 (14.3)	

Bold values are the statistically significant differences.

**Table 6:** The level of compliance with disinfection and sterilization practices (*N* = 175).

Characteristics	Disinfection and sterilization level of compliance			Value of the *χ*^2^ test *P* value
	Poor *N* (%)	Intermediate *N* (%)	Excellent *N* (%)	
Age (years)				
<30	50 (45.5)	26 (23.6)	34 (30.9)	4.41
30–39	17 (39.5)	9 (20.9)	17 (39.5)	0.353
≥40	7 (31.8)	9 (40.9)	6 (27.3)	
Gender				
Male	23 (37.1)	21 (33.9)	18 (29.0)	3.890
Female	51 (45.1)	23 (20.4)	39 (34.5)	0.143
Educational level				
Diploma or less	21 (43.8)	15 (31.3)	12 (25.0)	2.179
Bachelor or higher	53 (41.7)	29 (22.8)	45 (35.4)	0.336
Practice type				
Private clinics	19 (28.4)	23 (34.3)	25 (37.3)	10.207
Academic institutions	33 (54.1)	12 (19.7)	16 (26.2)	**0.037**
Public hospital	22 (46.8)	9 (19.1)	16 (34.0)	
Years as a dental radiographer				
<5	28 (39.4)	11 (15.5)	32 (45.1)	11.257
5–10	37 (45.7)	24 (29.6)	20 (24.7)	**0.024**
>10	9 (39.1)	9 (39.1)	5 (21.7)	
Hours worked for week				
<30	14 (38.9)	10 (27.8)	12 (33.3)	7.25
30–40	48 (50.5)	19 (20.0)	28 (29.5)	0.123
>40	12 (27.3)	15 (34.1)	17 (38.6)	
Number of patients per day				
≤10	26 (37.1)	23 (32.9)	21 (30.0)	8.75
11–20	30 (53.6)	12 (21.4)	14 (25.0)	0.068
>20	18 (36.7)	9 (18.4)	22 (44.9)	

Bold values are the statistically significant differences.

**Table 7:** The level of compliance with the use of surface barriers (*N* = 175).

Characteristics	Use of surface barriers level of compliance			Value of the *χ*^2^ test *P* value
	Poor *N* (%)	Intermediate *N* (%)	Excellent *N* (%)	
Age (years)				
<30	62 (56.4)	12 (10.9)	36 (32.7)	9.31
30–39	16 (37.2)	12 (27.9)	15 (34.9)	**0.050**
≥40	14 (63.6)	3 (13.6)	5 (22.7)	
Gender				
Male	36 (58.1)	9 (14.5)	17 (27.4)	1.233
Female	56 (49.6)	18 (15.9)	39 (34.5)	0.540
Educational level				
Diploma or less	26 (54.2)	5 (10.4)	17 (35.4)	1.35
Bachelor or higher	66 (52.0)	22 (17.3)	39 (30.7)	0.509
Practice type				
Private clinics	28 (41.8)	19 (28.4)	20 (29.9)	17.18
Academic institutions	37 (60.7)	7 (11.5)	17 (27.9)	**0.002**
Public hospital	27 (57.4)	1 (2.1)	19 (40.4)	
Years as a dental radiographer				
<5	29 (40.8)	13 (18.3)	29 (40.8)	9.23
5–10	17 (73.9)	11 (13.6)	24 (29.6)	0.056
>10	46 (56.8)	3 (13.0)	3 (13.0)	
Hours worked for week				
<30	22 (61.1)	2 (5.6)	12 (33.3)	7.875
30–40	53 (55.8)	17 (17.9)	25 (26.3)	0.096
>40	17 (38.6)	8 (18.2)	19 (43.2)	
Number of patients per day				
≤10	42 (60.0)	12 (17.1)	16 (22.9)	10.52
11–20	29 (51.8)	11 (19.6)	16 (28.6)	**0.032**
>20	21 (42.9)	4 (8.2)	24 (49.0)	

Bold values are the statistically significant differences.

## Data Availability

The data used to support the findings of this study are available from the corresponding author upon request.
